# 15-deoxy-Δ^12, 14^-prostaglandin J_2_ enhances anticancer activities independently of VHL status in renal cell carcinomas

**DOI:** 10.1016/j.bbrep.2019.01.001

**Published:** 2019-02-14

**Authors:** Hiromi Koma, Yasuhiro Yamamoto, Tomonari Fujita, Tatsurou Yagami

**Affiliations:** a Department of Pharmaceutical Health Care, Faculty of Pharmaceutical Sciences, Himeji Dokkyo University, 2-1, kami-ohno 7-Chome, Himeji, Hyogo 670-8524, Japan; b Hyogo Prefectural Kobe High School, 1-5-1 Shironoshita-dori Nada-ku Kobe, Hyogo 657-0804, Japan

**Keywords:** Renal cell carcinoma, Von Hippel–Lindau, 15-deoxy-Δ^12, 14^-prostaglandin J_2_, Topoisomerase inhibitor

## Abstract

Renal cell carcinoma (RCC) is relatively resistant to chemotherapy and radiotherapy. Clear cell RCC (ccRCC) accounts for the majority of RCC, which have mutations or epigenetic silencing of the *von Hippel–Lindau* (*VHL*) gene. VHL-positive Caki-2 cells are killed by an endogenous anticancer substance, 15-deoxy-Δ^12, 14^-prostaglandin J_2_ (15d-PGJ_2_). The MTT reduction assay reflecting mitochondrial succinate dehydrogenase activity was employed for assessment of cell viability. We confirmed anticancer activities of camptothecin (topoisomerase I inhibitor), etoposide (topoisomerase II inhibitor), doxorubicin (topoisomerase II inhibitor) in VHL-positive Caki-2 cells. Combination of topoisomerase inhibitors with 15d-PGJ_2_ exhibited the synergistic effect in VHL-positive Caki-2 cells. However, 15d-PGJ_2_ did not increase cytotoxicities of topoisomerase inhibitors on VHL-negative 786-O cells. In addition, the 15d-PGJ_2_-enhanced antitumor activity of topoisomerase inhibitors was detected in neither VHL-positive nor VHL-negative RCC4 cells. Our finding indicated that 15d-PGJ_2_ enhanced the antitumor activity of topoisomerase inhibitors independently of VHL.

## 1 Introduction

Renal cell carcinomas (RCCs) account for approximately 2% of adult carcinomas and arise from renal tubular epithelial cells that encompasses 85% of all primary renal neoplasms. RCCs are classified into several types such as clear cell RCC (ccRCC) accounting for the majority of RCC [1], papillary RCC and chromophobe RCC. The common genes involved in the pathogenesis of ccRCC include *von Hippel–Lindau* (*VHL*) [2]. VHL can be altered and transmitted in an autosomal dominant fashion (VHL disease) or in a sporadic manner. Despite extensive evaluation of many different treatment modalities, advanced metastatic RCC remains highly resistant to radiotherapy and chemotherapy [3].

To overcome the resistance of RCCs to chemotherapy, we have studied combinations of chemotherapy with anti-cancer agents. Responsiveness of RCCs such as VHL-positive Caki-2 cells for conventional anticancer agents such as camptothecin (CPT), etoposide (VP-16) and doxorubicin (DOX) was lower than that of other types of cancer such as Hela cells [4], [5], [6], [7], [8], [9]. CPT is a DNA topoisomerase I inhibitor, whereas VP-16 and DOX are DNA topoisomerase II inhibitors. Previously, we have reported that the anti-tumor activity of CPT was increased by 15-deoxy-Δ^12,14^-prostaglandin J_2_ (15d-PGJ_2_), which is an endogenous anticancer agent [7]. Although synergistic effect of 15d-PGJ_2_ and VP-16 on Caki-2 cells could not be detected in the absence of serum [7], 15d-PGJ_2_ elevated the anti-tumor activity of VP-16 in the presence of serum [8]. Peroxisome proliferator-activated receptor-γ (PPARγ) is a nuclear receptor for 15d-PGJ_2_ [10], [11]. However, it does not mediate the cytotoxicity of 15d-PGJ_2_ in RCCs [12], [13]. Furthermore, synergistic toxicities of 15d-PGJ_2_ with topoisomerase inhibitors were also independent from PPARγ.

In cancer, the phosphoinositide 3-kinase (PI3K)/Akt and mTOR pathway is activated via multiple mechanisms [14]. Since the PI3K signaling is hyperactivated in RCCs, this pathway is one of targeted therapies [15]. 15d-PGJ_2_ inhibits proliferation of primary neurons [16], [17], [18] and neuroblastoma x DRG neuron hybrid cell line N18D3 [19] via down-regulating PI3K/Akt pathway. Previously, we have reported that the PI3K/Akt signaling mediated the cytotoxicity of 15d-PGJ_2_ [13]. Although a PI3K inhibitor mimicked the cytotoxicity of 15d-PGJ_2_, it was not involved in the synergistic effect of 15d-PGJ_2_ on the anti-tumor activity of DOX [9]. VHL has been reported to be involved in the synergy between 5-aza-2′-deoxycytidine and paclitaxel [20]. To ascertain whether VHL was involved in the synergy between topoisomerase inhibitors and 15d-PGJ_2_, we compared the synergism of anti-cancer agents with 15d-PGJ_2_, in VHL-positive cell lines (Caki-2, ACHN and RCC4 (+)) and VHL-negative cell lines (786-O cells and RCC4(-)).

## 2 Materials and methods

### 2.1 Cell lines and cell culture

Caki-2, ACHN and RCC4(+) cells are the VHL-positive human RCC cell lines. 786-O and RCC4(-) cells are the VHL-negative human RCC cell lines. 786-O, ACHN, and Caki-2 cells were purchased from Summit Pharmaceuticals International (Tokyo, Japan). RCC4(+) and RCC4(-) cells were obtained from KAC Co. Ltd. (Kyoto, Japan). The Caki-2 and 786-O cells were routinely cultured in RPMI-1640 medium supplemented with 10% fetal bovine serum, 50 mg/ml penicillin G and 50 mg/ml streptomycin (Invitrogen, Tokyo, Japan), at 37 °C in a 5% CO_2_–95% room air. The RCC4(+) and RCC4(-) cells were routinely cultured in Dulbecco's Modified Eagle's Medium (DMEM) supplemented with 10% fetal bovine serum, 50 mg/ml penicillin G and 50 mg/ml streptomycin (Invitrogen, Tokyo, Japan), at 37 °C in a 5% CO_2_–95% room air.

### 2.2 Reagents

15d-PGJ_2_ (ab141717) was obtained from Abcam (Tokyo, Japan). Camptothecin (CPT), doxorubicin (DOX), etoposide (VP-16) and RPMI-1640 were purchased from FUJIFILM Wako Pure Chemical Corporation, Ltd. (Osaka, Japan). 3-(4,5-dimethylthiazol-2-yl)-2,5-diphenyl tetrazolium bromide dye (MTT) was purchased from Dojindo Laboratories (Kumamoto, Japan). The protein concentration was measured using the bicinchoninic acid (BCA) protein assay reagent obtained from Takara (Shiga, Japan). The principle of the assay is based on monovalent copper ions interact with a BCA reagent to form a violet reactive complex, which shows a strong absorbance at 562 nm. The peptide bonds in the protein reduce copper ions from Cu^2+^ to Cu^+^. The quantity of reduced Cu^2+^ is proportional to the amount of protein. The sample solution was added the BCA reagent and incubated at 37 °C for 30 min. The colorimetric variations were analyzed by spectrophotometer (iMark Microplate Reader, Bio Rad Laboratories, Hercules, CA, USA) at 562 nm. The experiments were analyzed in triplicate.

### 2.3 Cell viability analysis

MTT reduction assay reflecting mitochondrial succinate dehydrogenase activity was employed. The cells were seeded on a 96-well tissue culture plate at 10,000 cells/cm^2^ and incubated for 24 h prior to drug exposure. The cells were incubated with 15d-PGJ_2_ and doxorubicin at the indicated concentrations. After 20 h or 24 h, the cells were incubated with MTT solution (0.1 mg/ml in phosphate-buffered saline) for an additional 3 h at 37 °C. The MTT solution was then aspirated off. To dissolve the formazan crystals formed in viable cells, 100 μl dimethyl sulfoxide was added to each well. Absorbance was measured at 570 nm using a spectrophotometer (iMark Microplate Reader, Bio-Rad Laboratories, Hercules, CA, USA).

### 2.4 Statistical analysis

Data are given as means ± SE (n = numbers of observations). We performed two experiments at least on different days, and confirmed their reproducibility. We analyzed　observations obtained on the same day, and presented the typical experimental results among independent ones on different days to minimize experimental errors. Data were statistically analyzed with the Student's *t*-test for comparison with the control group. Data on various drugs were statistically analyzed by two-way ANOVA followed by Dunnett's test for comparison between the groups.

## 3 Results

### 3.1 Effects of 15d-PGJ_2_ on the anti-cancerous agents in Caki-2 cells

Previously, we have reported synergistic effects of 15d-PGJ_2_ and topoisomerase inhibitors [7], [8], [9]. In VHL-positive Caki-2 cells, CPT, VP-16, DOX and 15d-PGJ_2_ induced cell death via apoptosis in a concentration-dependent manner. At their sublethal concentrations, caspase-3 activity was markedly elevated by the combination of 15d-PGJ_2_ and topoisomerase inhibitors. As shown in supplemental data 1, we confirmed that 15d-PGJ_2_ significantly enhanced the cytotoxicity of topoisomerase inhibitors.

### 3.2 Effects of 15d-PGJ_2_ on the anti-cancerous agents in 786-O cells

**Fig. 1:**
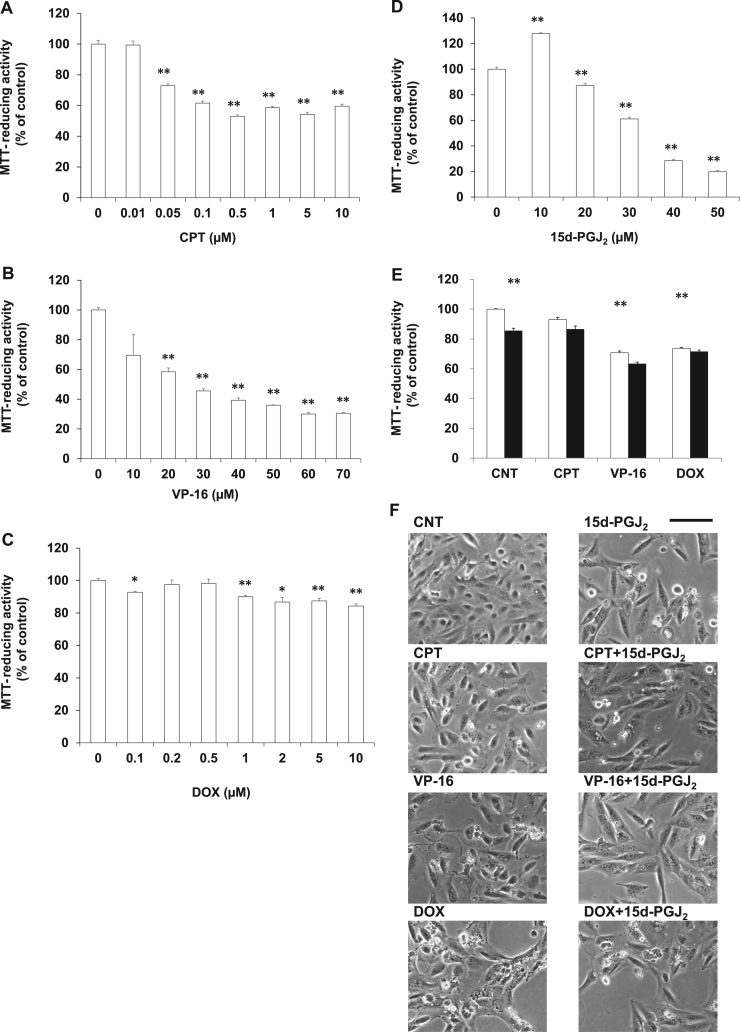
Effects of 15d-PGJ_2_ on the anti-cancerous activities of topoisomerase inhibitors in 786-O cells**.** 786-O cells were treated with CPT (A), VP-16 (B), DOX (C) or 15d-PGJ_2_ (D) at the indicated concentrations for 24 h. Cell viabilities were determined by MTT-reducing activity. Data are expressed as means ± SE. (n = 3). *P < 0.05, compared with control, **P < 0.01, compared with control. (E) 786-O cells were treated with 1 μM CPT, 5 μM VP-16 or 10 μM DOX in the absence (open column) or presence (closed column) of 20 μM 15d-PGJ_2_ for 24 h. Cell viabilities were determined by MTT-reducing activity. Data are expressed as means ± SE. (n = 6). *P < 0.05, compared with control, **P < 0.01, compared with control. (F) 786-O cells were treated with 1 μM CPT, 5 μM VP-16 or 10 μM DOX in the absence or presence of 20 μM 15d-PGJ_2_ for 24 h. Morphologies were photographed by phase contrast. Scale bar = 100 µm.

To ascertain whether topoisomerase inhibitors and 15d-PGJ_2_ synergistically exhibit the pharmacological effects on the VHL-negative ccRCC as well as the VHL-positive ccRCC, Caki-2 cells, 786-O cells were used as the VHL-negative ccRCC. In 786-O cells, CPT (Fig. 1A), VP-16 (Fig. 1B) or DOX (Fig. 1C) induced cell death in a concentration-dependent manner. As shown in Fig. 1D, 15d-PGJ_2_ also induced cell death in a concentration-dependent manner. We evaluated the synergism of 0.05 μM CPT, 5 μM VP-16 or 10 μM DOX with 20 μM 15d-PGJ_2_ by MTT-reducing activity (Fig. 1E). Although 15d-PGJ_2_ decreased the MTT-reducing activity and the cell number (Fig. 1F) significantly, it did not increase anticancer activities of the above three drugs significantly. At 10 μM, CPT, VP-16 and DOX degenerated morphologies slightly, moderately and severely, respectively. However, these degenerative morphologies were not deteriorated by 15d-PGJ_2_ (Fig. 1F). Although various concentrations of drugs were tested, we have not yet succeeded in detecting synergistic effect of CPT, VP-16 and DOX with 15d-PGJ_2_. Thus, the synergism of topoisomerase inhibitors and 15d-PGJ_2_ was not detected in the VHL-negative ccRCC, suggesting that VHL might be involved in the combinational effect of topoisomerase inhibitors and 15d-PGJ_2_.

### 3.3 Effects of 15d-PGJ_2_ on the anti-cancerous agents in RCC4 (-) cells

**Fig. 2:**
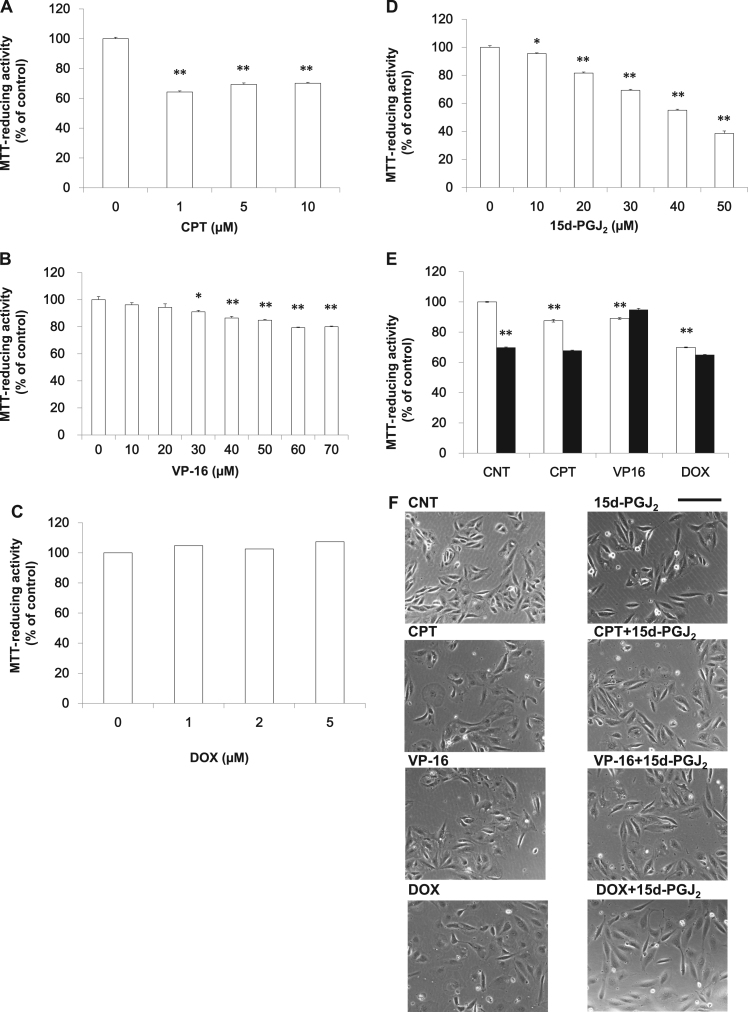
Effects of 15d-PGJ_2_ on the anti-cancerous activities of topoisomerase inhibitors in RCC4 (-) cells**.** RCC4 (-) cells were treated with CPT (A), VP-16 (B), DOX (C) or 15d-PGJ_2_ (D) at the indicated concentrations for 24 h. Cell viabilities were determined by MTT-reducing activity. Data are expressed as means ± SE. (n = 6). *P < 0.05, compared with control, **P < 0.01, compared with control. (E) RCC4 (-) cells were treated with 0.05 μM CPT, 20 μM VP-16 or 1 μM DOX in the absence (open column) or presence (closed column) of 20 μM 15d-PGJ_2_ for 24 h. Cell viabilities were determined by MTT-reducing activity. Data are expressed as means ± SE. (n = 6). **P < 0.01, compared with control. (F) RCC4 (-) cells were treated with 0.05 μM CPT, 20 μM VP-16 or 1 μM DOX in the absence or presence of 20 μM 15d-PGJ_2_ for 24 h. Morphologies were photographed by phase contrast. Scale bar = 100 µm.

To confirm the result obtained from the VHL-negative 786-O cells, RCC4 (-) was used as another VHL-negative RCC. In RCC4 (-) cells, CPT (Fig. 2A), VP-16 (Fig. 2B) or DOX (Fig. 2C) induced cell death in a concentration-dependent manner. As shown in Fig. 2D, 15d-PGJ_2_ induced cell death in a concentration-dependent manner. We evaluated the synergism of 0.05 μM CPT, 20 μM VP-16 or 1 μM DOX with 20 μM 15d-PGJ_2_ by the MTT-reducing activity (Fig. 2E) and the morphological criteria (Fig. 2F). CPT and VP-16 decreased the MTT-reducing activity to around 90% of control, whereas 15d-PGJ_2_ and DOX did it to around 70% of control. 15d-PGJ_2_ did not increase the anticancer activities of the above three drugs. Morphologies of RCC4(-) were similar to those of RCC4(+). Although cell densities appeared to be reduced by the four drugs, morphologies of RCC4(+) were not deteriorated by them significantly. Although various concentrations of drugs were tested, we have not yet detected synergistic effect of CPT, VP-16 and DOX with 15d-PGJ_2_. Thus, 15d-PGJ_2_ did not increase the anti-cancerous activities of topoisomerase inhibitors in the two VHL-negative RCCs.

### 3.4 Effects of 15d-PGJ_2_ on the anti-cancerous agents in RCC4 (+) cells

**Fig. 3:**
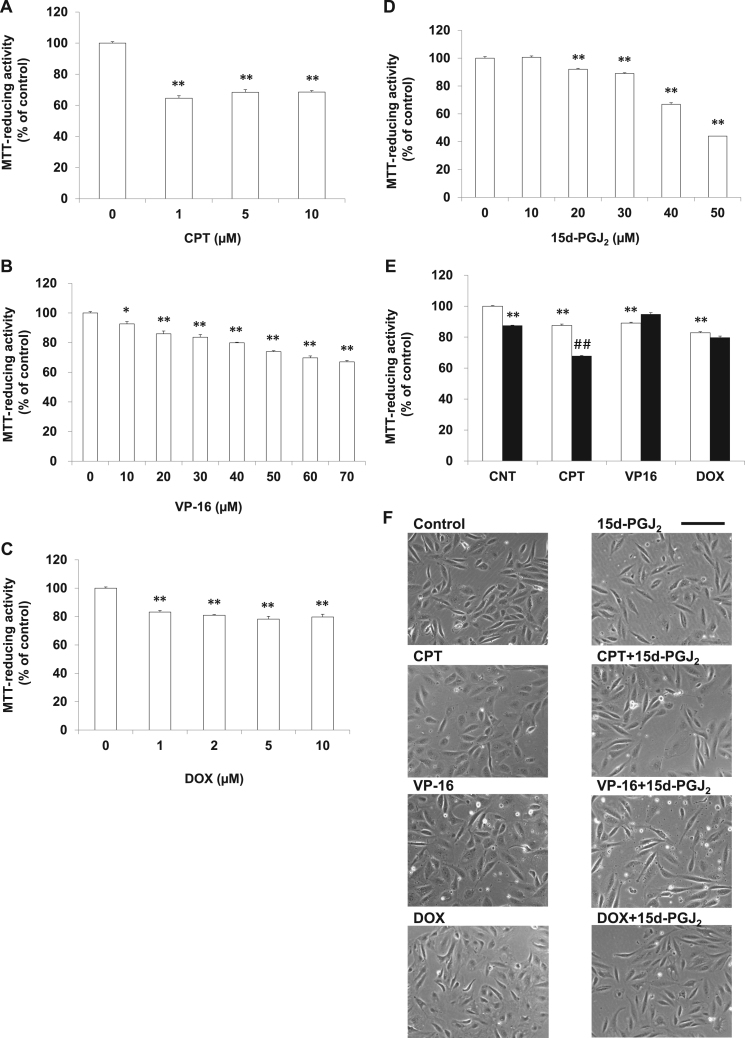
Effects of 15d-PGJ_2_ on the anti-cancerous activities of topoisomerase inhibitors in RCC4 (+) cells. RCC4 (+) cells were treated with CPT (A), VP-16 (B), DOX (C) or 15d-PGJ_2_ (D) at the indicated concentrations for 24 h. Cell viabilities were determined by MTT-reducing activity. Data are expressed as means ± SE. (n = 3). *P < 0.05, compared with control, **P < 0.01, compared with control. (E) RCC4 (+) cells were treated with 0.05 μM CPT, 20 μM VP-16 or 1 μM DOX in the absence (open column) or presence (closed column) of 20 μM 15d-PGJ_2_ for 24 h. Cell viabilities were determined by MTT-reducing activity. Data are expressed as means ± SE. (n = 6). **P < 0.01, compared with control. ^##^P < 0.01, compared with each topoisomerase inhibitor alone. (F) RCC4 (+) cells were treated with 0.05 μM CPT, 20 μM VP-16 or 1 μM DOX in the 0.05 absence or presence of 20 μM 15d-PGJ_2_ for 24 h. Morphologies were photographed by phase contrast. Scale bar = 100 µm.

To confirm the plausible involvement of VHL in the combinational effect of topoisomerase inhibitors and 15d-PGJ_2_, RCC4(+) and RCC4(-) were evaluated as another VHL-positive and VHL-negative RCCs, respectively. In RCC4(+) cells, CPT (Fig. 3A), VP-16 (Fig. 3B) or DOX (Fig. 3C) induced cell death in a concentration-dependent manner. As shown in Fig. 3D, 15d-PGJ_2_ induced cell death in a concentration-dependent manner. We evaluated the synergism of 0.05 μM CPT, 20 μM VP-16 or 1 μM DOX with 20 μM 15d-PGJ_2_ by the MTT-reducing activity (Fig. 3E) and the morphological criteria (Fig. 3F). Although these four anti-cancer agents did not alter the morphology of RCC4(+) clearly, they exhibited cytotoxicities slightly, but significantly. 15d-PGJ_2_ increased the anticancer activity of CPT additively, whereas it did not those of the two topoisomerase II inhibitors. In spite of testing various concentrations of drugs, we have not yet succeeded in detecting synergistic effect of CPT, VP-16 and DOX with 15d-PGJ_2_. Contrary to the result obtained from the VHL-positive Caki-2 cells, 15d-PGJ_2_ did not enhanced the anti-cancer activity of topoisomerase inhibitors synergistically in the VHL-positive RCC4(+) cells.

### 3.5 Effects of 15d-PGJ_2_ on the anti-cancerous agents in ACHN cells

**Fig. 4:**
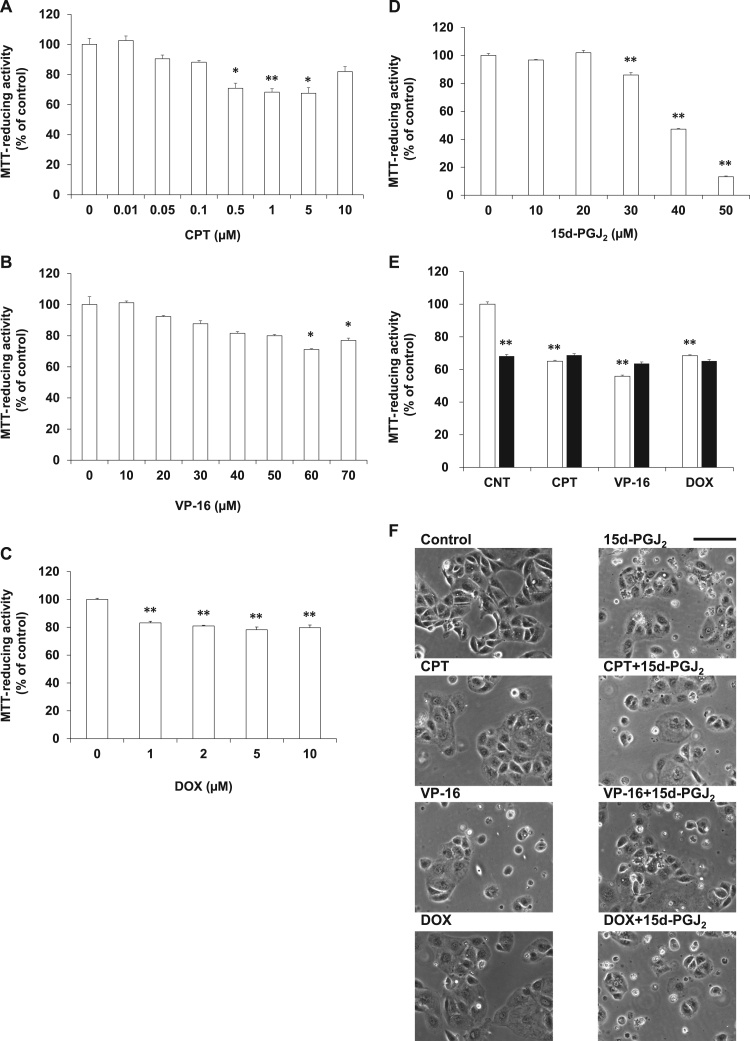
Effects of 15d-PGJ_2_ on the anti-cancerous activities of topoisomerase inhibitors in ACHN cells. ACHN cells were treated with CPT (A), VP-16 (B), DOX (C) or 15d-PGJ_2_ (D) at the indicated concentrations for 24 h. Cell viabilities were determined by MTT-reducing activity. Data are expressed as means ± SE. (n = 3). *P < 0.05, compared with control, **P < 0.01, compared with control. (E) ACHN cells were treated with 0.5 μM CPT, 50 μM VP-16 or 0.5 μM DOX in the absence (open column) or presence (closed column) of 30 μM 15d-PGJ_2_ for 24 h. Cell viabilities were determined by MTT-reducing activity. Data are expressed as means ± SE. (n = 6). **P < 0.01, compared with control. ^##^P < 0.01, compared with each topoisomerase inhibitor alone. (F) ACHN cells were treated with 0.5 μM CPT, 50 μM VP-16 or 0.5 μM DOX in the absence or presence of 30 μM 15d-PGJ_2_ for 24 h. Morphologies were photographed by phase contrast. Scale bar = 100 µm.

To confirm the plausible involvement of VHL in the combinational effect of topoisomerase inhibitors and 15d-PGJ_2_, ACHN cells were evaluated as another VHL-positive RCCs. In ACHN cells, CPT (Fig. 4A), VP-16 (Fig. 4B) or DOX (Fig. 4C) induced cell death in a concentration-dependent manner. As shown in Fig. 4D, 15d-PGJ_2_ induced cell death in a concentration-dependent manner. We evaluated the synergism of 0.5 μM CPT, 50 μM VP-16 or 0.5 μM DOX with 30 μM 15d-PGJ_2_ by the MTT-reducing activity (Fig. 4E) and the morphological criteria (Fig. 4F). Although these four anti-cancer agents did not alter the morphology of ACHN clearly, they exhibited cytotoxicities slightly, but significantly. 15d-PGJ_2_ did not increase the anticancer activities of the three topoisomerase inhibitors. In spite of testing various concentrations of drugs, we have not yet succeeded in detecting synergistic effect of CPT, VP-16 and DOX with 15d-PGJ_2_. Contrary to the result obtained from the VHL-positive Caki-2 cells, 15d-PGJ_2_ did not enhanced the anti-cancer activity of topoisomerase inhibitors synergistically in the VHL-positive ACHN cells.

## 4 Discussion

Previously, we have reported 15d-PGJ_2_ as the endogenous anticancer agent in Caki-2 cells [7], [8], [9]. Neither nuclear receptor PPARγ nor membrane receptor CRTH2 mediate the cytotoxicity of 15d-PGJ_2_. 15d-PGJ_2_ also exhibited anti-cancerous effects in other RCCs such as 786-O, RCC4(-), RCC4(+) and ACHN. Similarly to Caki-2 cells, RCC4(+), RCC4(-) and 786-O cells are small polygonal growing cells forming small aggregates. 15d-PGJ_2_ targets the cytoskeleton protein, actin, resulting in alteration of cell morphologies [21]. Actin is one of adapter proteins, which mediates the intracellular domain of integrin bind to the cytoskeleton. Since this integrin-adapter protein (actin) -cytoskeleton complex forms the basis of a focal adhesion, it was likely that 15d-PGJ_2_ increased protrusions and made focal adhesion clear.

In the present study, we confirmed that 15d-PGJ_2_ enhanced the anti-tumor activity of topoisomerase I inhibitor CPT (plant alkaloids) [7], topoisomerase II inhibitors VP-16 (plant alkaloids) [8] and DOX (antibiotics) [9]. Morphological alterations could not be detected at sublethal concentrations of CPT (1 μM), VP-16 (70 μM) and DOX (1 μM) in Caki-2 cells. Rounding cell shape and shrinking cell bodies were markedly increased by the combination of these topoisomerase inhibitors with 15d-PGJ_2_. However, PPARγ was not involved in the enhancement of 15d-PGJ_2_ on the anti-tumor activities of topoisomerase inhibitors [7], [8], [9]. Capase-3 is significantly activated by either 15d-PGJ_2_ alone or each topoisomerase inhibitor alone. The capase-3 activity is elevated synergistically by their combination [7], [8], [9]. Thus, 15d-PGJ_2_ potentiated the pharmacological effect of topoisomerase inhibitors in Caki-2 cells.

VHL is a tumor suppressor protein and localized in the nucleus or cytoplasm. VHL forms a protein complex, which determines ubiquitin-dependent proteolysis of large cellular proteins. When normal oxygen levels are present, the complex binds to, and targets, α subunits of hypoxia-inducible factors (HIF) 1 and 2 for ubiquitin-mediated degradation of protein [22]. Caki-2 cell line has been established from a primary tumor of the kidney. Although it has been primarily defined as the ccRCC cell line, it expresses wild-type pVHL. However, a low expression of HIF-1α and no expression of HIF-2α is detected in Caki-2 cell line [23]. 786-O has many characteristics of ccRCC and is defective in VHL expression, as it harbors mutated VHL [24]. In 786-O cells, cytotoxicities of topoisomerase I and II inhibitors were detected. Although 15d-PGJ_2_ induced cell death in 786-O cells, it did not potentate the anti-tumor activity of topoisomerase inhibitors. Another cell line is RCC4, a VHL mutant derived from a primary tumor widely used as a model for VHL-dependent mechanisms, with a commercially available counterpart cell line with restored wild-type gene [22]. Cytotoxicities of CPT, VP-16 and DOX were detected in the two RCC4(-) and RCC4(+) cells. However, 15d-PGJ_2_ enhanced the anti-tumor activity of these topoisomerase inhibitors in neither RCC4(-) nor RCC4(+) cells. Thus, the pharmacological synergism of 15d-PGJ_2_ and topoisomerase inhibitors were not depend on the state of VHL.

Previously, we have reported that the PI3K/Akt signaling played an important role in the cytoprotection and proliferation of RCCs [13]. 15d-PGJ_2_ markedly decreased the phosphorylation of Akt. The Akt inhibitor showed cytotoxicity with a low IC_50_ value, suggesting that 15d-PGJ_2_ exerted cytotoxicity via the inactivation of Akt. The PI3K inhibitor mimicked the anti-tumor activity of 15d-PGJ_2_. However, we could not detect the synergistic effect between DOX and PI3K inhibitor. In addition, the PI3K inhibitor did not enhanced cytotoxicities of another topoisomerase II inhibitor, etoposide, and a topoisomerase inhibitor I, camptothecin. Neither PPARγ nor PI3K was involved in the 15d-PGJ_2_-enhanced chemosensitivity of Caki-2 cells to topoisomerase inhibitors. Further studies are required to identify targets for 15d-PGJ_2_, which reduces the chemoresistance of topoisomerase inhibitors.

## 5 Conclusion

In the present study, we ascertained whether VHL was involved in the synergy between topoisomerase inhibitors and 15d-PGJ_2_ or not. We demonstrated that 15d-PGJ_2_ enhanced anticancer activities independently of VHL status in renal cell carcinomas.

## Appendix A Supplementary material

Supplementary material Supplemental data 1. 15d-PGJ_2_ enhanced anti-cancerous activities of topoisomerase inhibitors in Caki-2 cells. Caki-2 cells were treated with control (CNT), 1 μM camptothecin (CPT), 40 μM etoposide (VP-16) and 1 μM doxorubicin (DOX) in the absence (open column) or presence (closed column) of 20 μM 15d-PGJ_2_ for 24 h. Cell viabilities were determined by MTT-reducing activity. Data are expressed as means ± SE. (n = 6). **P < 0.01, compared with control. ^##^P < 0.01, compared with 15d-PGJ_2_ alone or drug alone.
