# Supplementary material for: 15-deoxy-Δ12, 14-prostaglandin J2 enhances anticancer activities independently of VHL status in renal cell carcinomas
Source: Biochem Biophys Rep. 2019 Feb 14;18:100608. doi: 10.1016/j.bbrep.2019.01.001 (PMC6377412; doi:10.1016/j.bbrep.2019.01.001)
Supplement: Supplementary file 1 — Supplementary material Supplemental data 1. 15d-PGJ2 enhanced anti-cancerous activities of topoisomerase inhibitors in Caki-2 cells. Caki-2 cells were treated with control (CNT), 1 μM camptothecin (CPT), 40 μM etoposide (VP-16) and 1 μM doxorubicin (DOX) in the absence (open column) or presence (closed column) of 20 μM 15d-PGJ2 for 24 h. Cell viabilities were determined by MTT-reducing activity. Data are expressed as means ± SE. (n = 6). **P < 0.01, compared with control. ##P < 0.01, compared with 15d-PGJ2 alone or drug alone. [file mmc1.docx]

**Supplemental data 1. 15d-PGJ_2_ enhanced anti-cancerous activities of topoisomerase inhibitors in Caki-2 cells.**

Caki-2 cells were treated with control (CNT), 1 μM camptothecin (CPT), 40 μM etoposide (VP-16) and 1 μM doxorubicin (DOX) in the absence (open column) or presence (closed column) of 20 μM 15d-PGJ_2_ for 24 h. Cell viabilities were determined by MTT-reducing activity. Data are expressed as means ± SE. (n = 6). **P < 0.01, compared with control. ##P < 0.01, compared with 15d-PGJ_2_ alone or drug alone.
