# Supplementary material for: 15-deoxy-Δ12, 14-prostaglandin J2 enhances anticancer activities independently of VHL status in renal cell carcinomas
Source: Biochem Biophys Rep. 2019 Feb 14;18:100608. doi: 10.1016/j.bbrep.2019.01.001 (PMC6377412; doi:10.1016/j.bbrep.2019.01.001)
Supplement: Supplementary file 2 — Supplementary material [file mmc2.docx]

**Conflict of Interest** The authors declare no conflict of interest.
